# A systematic review of transmission dynamic studies of methicillin-resistant *Staphylococcus aureus* in non-hospital residential facilities

**DOI:** 10.1186/s12879-018-3060-6

**Published:** 2018-04-18

**Authors:** Kin On Kwok, Jonathan M. Read, Arthur Tang, Hong Chen, Steven Riley, Kai Man Kam

**Affiliations:** 1 The Jockey Club School of Public Health and Primary Care, The Chinese University of Hong Kong, Shatin, Hong Kong, Special Administrative Region of China; 2 Stanley Ho Centre for Emerging Infectious Diseases, The Chinese University of Hong Kong, Shatin, Hong Kong, Special Administrative Region of China; 3 Shenzhen Research Institute of the Chinese University of Hong Kong, Shenzhen, China; 4 Centre for Health Informatics Computing and Statistics, Lancaster Medical School, Faculty of Health and Medicine, Lancaster University, Lancaster, UK; 5 Institute of Infection and Global Health, The Farr Institute@HeRC, University of Liverpool, Liverpool, UK; 6 Department of Software, Sungkyunkwan University, Seoul, South Korea; 7 Centre for Health Protection, Hong Kong, Hong Kong, Special Administrative Region of China; 8 MRC Centre for Outbreak Analysis and Modelling, Department for Infectious Disease Epidemiology, Imperial College London, London, UK

**Keywords:** Methicillin-resistant *Staphylococcus aureus*, MRSA, Transmission models, Non-hospital, Residential

## Abstract

**Background:**

Non-hospital residential facilities are important reservoirs for MRSA transmission. However, conclusions and public health implications drawn from the many mathematical models depicting nosocomial MRSA transmission may not be applicable to these settings. Therefore, we reviewed the MRSA transmission dynamics studies in defined non-hospital residential facilities to: (1) provide an overview of basic epidemiology which has been addressed; (2) identify future research direction; and (3) improve future model implementation.

**Methods:**

A review was conducted by searching related keywords in PUBMED without time restriction as well as internet searches via Google search engine. We included only articles describing the epidemiological transmission pathways of MRSA/community-associated MRSA within and between defined non-hospital residential settings.

**Results:**

Among the 10 included articles, nursing homes (NHs) and correctional facilities (CFs) were two settings considered most frequently. Importation of colonized residents was a plausible reason for MRSA outbreaks in NHs, where MRSA was endemic without strict infection control interventions. The importance of NHs over hospitals in increasing nosocomial MRSA prevalence was highlighted. Suggested interventions in NHs included: appropriate staffing level, screening and decolonizing, and hand hygiene. On the other hand, the small population amongst inmates in CFs has no effect on MRSA community transmission. Included models ranged from system-level compartmental models to agent-based models. There was no consensus over the course of disease progression in these models, which were mainly featured with NH residents /CF inmates/ hospital patients as transmission pathways. Some parameters used by these models were outdated or unfit.

**Conclusions:**

Importance of NHs has been highlighted from these current studies addressing scattered aspects of MRSA epidemiology. However, the wide variety of non-hospital residential settings suggest that more work is needed before robust conclusions can be drawn. Learning from existing work for hospitals, we identified critical future research direction in this area from infection control, ecological and economic perspectives. From current model deficiencies, we suggest more transmission pathways be specified to depict MRSA transmission, and further empirical studies be stressed to support evidence-based mathematical models of MRSA in non-hospital facilities. Future models should be ready to cope with the aging population structure.

**Electronic supplementary material:**

The online version of this article (10.1186/s12879-018-3060-6) contains supplementary material, which is available to authorized users.

## Background

Methicillin-resistant *Staphylococcus aureus* (MRSA) often induces infections that are difficult to treat because of its ability to survive most antibiotics. The World Health Organization has recently listed MRSA as one of the priority pathogens posing threat to human health [1]. MRSA is endemic in Hong Kong [2] with occasional outbreaks in hospitals, resulting in bacteremia, pneumonia and surgical site infections. Empirical studies have shown that non-hospital residential facilities are important reservoirs for MRSA transmission. Facilities with individuals residing for a substantial period, such as long term care facilities (LTCFs) and correctional facilities (CFs), are considered as at particularly high risk. Some studies reported that the MRSA colonization prevalence rate can be as high as 52% in nursing homes (NHs) in the United States (US) [3–5], significantly higher than that of 1.5% in the general population [6]. The MRSA acquisition rate was reported to be higher among residents in LTCFs than their stays in hospitals [7]. MRSA was also shown to be more prevalent in CFs than in the general community [8–11].

Hospitals and LTCFs are residential settings connected by the transfer of residents or patients [12, 13]. Transmission and epidemiological characteristics of MRSA in LTCFs may be different from those in hospitals, and may contribute extensively to community level transmission. The average length of stay (LOS) is longer for residents in NHs [14] and inmates in CFs [15] compared with patients in hospitals [16]. In fact, residents in NHs were shown to carry MRSA for a considerably long period of time: asymptomatic colonization could last more than 3 years [17, 18]. Several factors that contributed to the transmission mechanism characteristics of MRSA in non-hospital residential facilities being different from those in hospitals, including: (a) different interpersonal contact pattern; (b) different daily ward routines; (c) different health conditions of individuals in the facilities; and (d) facility-specific environmental factors.

Dynamic studies have been developed to study MRSA transmission within non-hospital residential settings. Models that explicitly represented how the risk of infection was related to the current number of infectious people [19] were useful for studying transmission dynamics, evaluating different infection control interventions, evaluating burden of infection as well as facilitating further understanding of LTCF epidemiology. Numerous mathematical models have been employed to depict MRSA transmission in hospital settings [20, 21], but the conclusions and public health implications drawn from these studies may not be applicable to non-hospital settings. In light of this, we conducted a systematic review of mathematical models for the MRSA transmission in non-hospital residential facilities. The aims of this review are to provide an overview of epidemiological understanding of MRSA transmission in non-hospital settings gained through mathematical modeling, to identify future research direction in this area and to improve future model implementation by addressing current models’ deficiencies.

## Methods

Non-hospital residential facilities were defined as non-hospital settings where individuals resided for a substantial period of time. Such facilities included CFs and LTCFs. Two types of CFs were jails and prisons. Jails were facilities holding individuals serving a short period (usually shorter than one year). Prisons were facilities that confine convicted individuals for a longer period. LTCFs were facilities that provided care to clients with medical services or daily needs over an extended period, including NHs and assisted living facilities (ALFs). NHs were mainly for elderly with medical needs. ALFs were for individuals from different ages and vulnerable groups who lived dependently, including children and people with long-term disability.

To identify studies for this review, an initial search using the PUBMED database in the field “Title/Abstract” was conducted using the following search terms:

1. MRSA OR “Methicillin-resistant *Staphylococcus aureus*” OR “Methicillin resistant *Staphylococcus aureus*” AND
2. dynamics OR agent-based OR “agent based” OR individual-based OR “individual based” OR mathematical OR Bayesian OR compartmental OR deterministic OR stochastic AND
3. “children care” OR “child care” OR jail OR prison OR custody OR correctional OR elderly OR nursing OR long-term care OR “long term care” OR “care home” OR boarding OR residential OR non-hospital OR “non hospital”

The search was further expanded by internet searches using the keywords “MRSA”, “transmission”, “dynamics”, “modelling” and “correctional” in Google search engine on 26 May 2017. Results were screened up to the fifth pages returned from the internet search.

Only articles depicting epidemiological transmission pathways of MRSA/community-associated MRSA within and between defined non-hospital residential settings were included in this review. Population-level studies were excluded. Included articles were summarized in terms of: study aims, countries for model inference, model types and forecast period, disease progression and transmission pathways characterized by the model, and technical model execution details (model assumptions, parameter values, and ways of parameterization).

Two authors (KOK, SR) screened the titles and abstracts of articles obtained from the initial search. To finalize articles included in this study, two authors (KOK, SR) read the full-text of the shortlisted publications and excluded articles that did not fit into the context for this review.

## Results

**Fig. 1:**
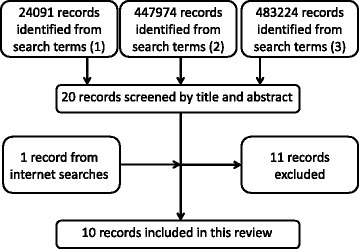
Flow diagram of present study. Nine articles out of 20 were shortlisted to be included in this study. One additional article was shortlisted by the ad hoc method. After final screening on the shortlisted publications, 10 articles were selected for review. Three modeled the intra-facility dynamics in NHs [22–24], three modeled the intra-facility dynamics in CFs [25–27], and four modeled patient inter-facility dynamics [28–31]

The search was performed in the PUBMED database on 23rd April 2017. Ten articles were identified from the PUBMED database and by internet searches (Fig. 1). Three studies focused on MRSA transmission dynamics within NHs [22–24], three focused on that associated with CFs [25–27], and four focused on that between facilities [28–31]. Seven aspects of basic epidemiology of MRSA in non-hospital residential settings and model frameworks of included studies were summarized.

### Role of health-care workers (HCWs) and residents in transmission mechanisms within NHs

Only one study represented HCWs and residents explicitly [22]. Potential outbreaks in NHs were considered more likely due to an imported MRSA colonized resident than due to contaminated HCWs [22]. The two studies conducted by *Batina* et al. did not consider HCWs in the transmission dynamics [23, 24]; only residents were considered. In the two models, residents were further classified based on their antibiotics use in the past three months. Residents with antibiotics exposure were more likely to acquire MRSA [24], increasing strain-specific MRSA prevalence [23]. None of these three studies considered HCWs as MRSA long-term carriers.

### Intervention strategies to control MRSA transmission within NHs

Appropriate staffing level for better infection control was suggested [22]. Given the presumed homogeneous mixing, increasing staff-to-residents ratio lowered the average number of contacts between HCWs and residents, resulting in a decrease of MRSA prevalence [22]. Screening and decolonizing colonized residents at admission were suggested by *Chamchod* et al. [22] and *Batina* et al. [23]. *Chamchod* et al. showed that MRSA would persist in NHs without screening and decolonization strategies [22]. *Batina* et al. highlighted the same strategies could theoretically eradicate MRSA in NHs [23]; and in practice these strategies could at least reduce the prevalence. Hand hygiene practices for residents and HCWs was suggested by *Chamchod* et al. [22].

### Persistence of MRSA within NHs

Long-term MRSA dynamics were used to investigate the possibility and magnitude of MRSA endemicity [22–24]. MRSA was endemic in NHs if no effective infection control intervention was implemented. However, MRSA could possibly be eradicated by strict screening and decolonization of colonized individuals at admission [22, 23]. *Batina* et al. concluded that an outbreak was unlikely to occur except in extraordinary conditions such as multifold increase in MRSA acquisition rate [23].

### Outbreak potential within CFs

*Kajita* et al. suggested that outbreak potential was small unless inmates were interned for at least 2 to 2.5 months [25]. *Beauparlant* et al. suggested that the increased inflow and outflow from high recidivism might lead to a sustained prevalence within CFs when re-offending rates were high [26].

### Impact of CF-community MRSA dynamics

*Kajita* et al. reported that within-jail MRSA transmission was sufficient to sustain continual outbreaks if there is a continuous inflow of colonized and infected individuals from the community [25]. *Beauparlant* et al. also suggested that outbreaks in jail were driven by community dynamics but CFs would not significantly affect community MRSA dynamics [26]. Despite findings that hospitals and prisons released a similar number of newly colonized individuals into community at an average rate [27], the small population size of prison made the impact of MRSA dynamics in community negligible [26].

### Impact of LTCF-hospital MRSA dynamics

*Barnes* et al. suggested that patient movement between hospitals and LTCFs contributed significantly to MRSA prevalence in LTCFs [28]. The endemic prevalence within LTCFs was shown to be positively associated with the MRSA prevalence of hospitals with which patients were shared. Patient transfers from hospitals were found to result in sustained increased in MRSA prevalence in LTCFs, particularly those with small population size and low residents’ turnover rates. *Lesosky* et al. studied MRSA transmission dynamics between NHs and hospitals in one metropolitan area [31]. They suggested the importance of NHs over hospitals in affecting the overall nosocomial MRSA prevalence when endemic prevalence was in almost all institutions. The increased transmission rate in a single NH caused a substantially higher percentage change to overall nosocomial MRSA prevalence than that caused by increased transmission rate in a single hospital when MRSA was endemic in all health-care settings. Changes in patient transfer rates or patterns among mainstream facilities did not significantly change the hospital MRSA prevalence [31]. *Lee* et al. stated that an MRSA outbreak in the largest NH increased the average MRSA prevalence in multiple hospitals with direct and indirect patient transfer for 6 months [29]. NHs should be considered as an important setting to implement hospital infection control strategy [29].

### Intervention strategies to control inter-facility MRSA transmission

Following the findings by *Lesosky* et al. [31], *Lee* et al. suggested that contact precaution use on known colonization carriers by reducing their contact rates in NHs could substantially reduce MRSA transmission in both NHs and hospitals [30].

### Modelling frameworks

Most reviewed studies depicted disease dynamics on a system level by compartmental models and were solved deterministically [22, 23, 25, 26]; three included stochastic solution [22, 23, 25], one used a Markov model [24] and one presented findings based on analytical results [27]. Some studies modeled disease dynamics with individual-level agent-based models [29, 30] and Monte Carlo simulation model [31], in which an individual was regarded as an agent with their own inter-facility movements and infection states. Hybrid models combining agent-based model (facilities are regarded as agents) and compartmental model (within-facility) were also used to describe the transmission dynamics on both system level and local level [28]. Table 1 summarizes key aspects of models featuring MRSA transmission dynamics in different settings, including model features and transmission pathways.

**Table 1:** Summary of key model specifications of reviewed models

Settings	Nursing Homes		
Articles	Chamchod et al. (2012) [22]	Batina et al. (2016a) [23]	Batina et al. (2016b) [24]
Aims	1. Study MRSA dissemination 2. Study persistence and prevalence of MRSA 3. Study intervention controls	1. Assess MRSA epidemic potential 2. Determine conditions at which USA300 and non-USA300 could be eliminated or reduced 3. Evaluate the impact of recent antibiotics exposure on MRSA prevalence and Ro	1. Predict long-term prevalence of USA300 and non-USA300 2. Assess the influence of potential risk factors on MRSA acquisition rates and average duration of colonization
Country (model inference)	Non-specific ^a^	Wisconsin, United States	Wisconsin, United States
Model			
Type^b^	Compartmental (deterministic); Markov process (stochastic)	Compartmental (deterministic) Markov process (stochastic)	Markov chain model
Forecast period	1200/2000/4000 days	20 years to 30 years	120 months
Disease progression			
Host	Residents	Residents	Residents
Vector	HCWs	Not applicable	Not applicable
States involved among hosts	Susceptible, Colonized	Susceptible, Colonized	Susceptible, Colonized
States involved among vectors	Decontaminated, contaminated	Not applicable	Not applicable
MRSA Strains involved	MRSA as a whole	USA300, non-USA300	USA300, non-USA300
Stratified by hosts’ recent antibiotics exposure	No	Yes	Yes
Transmission pathways			
Endogenous			
Residents to Residents	Yes	Yes	Not applicable ^d^
Residents to HCWs	Yes ^c^	No	Not applicable ^d^
HCWs to Residents	Yes ^c^	No	Not applicable ^d^
HCWs to HCWs	No ^c^	No	Not applicable ^d^
Exogenous			
Importation of colonized cases	Yes	Yes	Not applicable ^d^
Settings	Correctional facilities		
Articles	Hartley et al. (2006) [27]	Kajita et al. (2007) [25]	Beauparlant et al. (2016) [26] ^g^
Aims	1. Calculate the epidemiological weight^e^ of an institution / subpopulation	1. Assess outbreak severity 2. Determine the conditions and consequences of outbreaks 3. Design interventions to control outbreaks	1. Determine effect of community dynamics on MRSA dynamics in prisons 2. Determine the effect of recidivisms on disease dynamics
Country (model inference)	Non-specific ^f^	Los Angeles, United States	United States
Model			
Type^b^	Mathematical formula	Compartmental (deterministic, stochastic)	Compartmental (deterministic)
Forecast period	Not applicable	9 months	1000 days
Disease progression			
Host	Inmates	Inmates	Community, Inmates, Recidivists
States involved among hosts	Colonized, Non-colonized	Susceptible, Colonized, Infected	Susceptible, Infected
Strains involved	MRSA as a whole	CA-MRSA	MRSA as a whole
Stratified by hosts’ recent antibiotics exposure	No	No	No
Transmission pathways			
Endogenous			
Inmates to Inmates	Not applicable	Yes ^h^	Yes ^h,i^
Inmates to Staff	Not applicable	No	No
Staff to Inmates	Not applicable	No	No
Exogenous			
Importation of colonized cases	Not applicable	Yes	Yes^j^
Settings	Inter-facilities		
Articles	Barnes et al. (2011) [28]	Lesosky et al. (2011) [31]	Lee et al. (2013a) [29] Lee et al. (2013b) [30]
Aims	1. Predict long-term prevalence of facilities 2. Assess the effects of facility size, patient turnover and decolonization on MRSA prevalence	1. Determine how patient transfers affect MRSA transmission among patients in hospitals and NHs	[29]: 1. Quantify how MRSA prevalence in NHs affect those in hospitals [30]: 1. Compare different contact intervention strategies (no intervention VS only clinically apparent MRSA infections VS all MRSA carriers)
Country (model inference)	Non-specific ^f^	Non-specific ^k^	California, United States
Model			
Type^b^	Hybrid simulation model ^l^	Stochastic, discrete time Monte Carlo simulation model	Agent-based model
Forecast period	Not explicitly stated	365 days	[29]: 5 years after outbreak [30]: 5 years after outbreak implementing contact precautions
Facility involved	Hospitals, General LTCFs	Teaching hospitals (THs)^m^, Non-teaching hospitals (NTHs)^m^, NHs	Hospitals, NHs
Agent unit	Facility	Individual	Individual
Disease progression			
States involved	Susceptible, Persistently colonized, Colonized	Susceptible, Colonized/Infected	Susceptible, Colonized
Strains involved	MRSA as a whole	MRSA as a whole	MRSA as a whole
Transmission pathways			
Intra-facility			
Hospitals			
Patients to patients	Yes	Yes	Yes
Patients to HCWs	No	No	No
HCWs to HCWs	No	No	No
HCWs to patients	No	No	No
NHs/LTCFs			
Residents to residents	Yes	Yes	Yes
Residents to HCWs	No	No	No
HCWs to HCWs	No	No	No
HCWs to residents	No	No	No
Inter- facility (patient sharing)			
Hospitals to Hospitals	No	Yes	Yes
LTCFs/NHs to LTCFs/NHs	No	No	Yes
Hospitals to LTCFs/NHs	Yes	Yes	Yes
LTCFs/NHs to Hospitals	Yes	Yes^n^	Yes^n^

Remarks

^a^ The study model was parameterized with data from the Norway, Ireland, France, Italy, and United States

^b^ The choice of continuous time versus discrete time model is not generally important for these systems, because the number of individuals is small and allows the efficient simulation of both model types. In general, equation-based compartment models (CMs) and agent-based models (ABMs) produce similar, but not exact, results [77, 78]. CMs are easier to implement than AMBs, but they rely on parsimony assumptions for objects in the same compartment; whereas ABMs can feature the heterogeneity characteristics down to an individual level

^c^ HCWs were either contaminated or decontaminated but not MRSA carriers

^d^ Pathway was not explicitly stated in this model, the probability of individual MRSA colonization state at time t had reflected the present amount of colonized in the facilities and individual current MRSA status. The current state at time t was assumed to be only dependent on their states at time t-1

^e^ Epidemiological weight indicates the level of release of newly colonized individuals into the community from the facility at an average daily rate

^f^ The study model was parameterized with data from the United States

^g^ This article was retrieved from Google search engine. The other 9 articles were retrieved from PUBMED

^h^ No classification over direct (social mixing) and indirect (sharing towels and personal items) transmission pathways

^i^ Include both inmates and recidivists

^j^ There were imported cases into the prisons from community. However, instead of presenting this importation as admission probability, the authors integrated the overall disease dynamics in the community and among recidivists, and allowed flows between individuals of the same disease states, regardless of subpopulation

^k^ The study model was parameterized with data from Canada

^l^ Each facility was treated an agent, while the disease progression within a facility was featured by a compartmental model

^m^ Lesosky divided hospitals into 2 types: teaching (bigger in size) and non-teaching (smaller in size)

^n^ It includes temporary hospital admission where beds in NH would be kept for the agent until his/her return [29, 30] or for 30 days [31]

Different representations of disease progression were defined (Table 1). Three main transition states in MRSA were generally considered: uncolonized (U), colonized (C), and infected (I). Some studies further divided the colonized state into persistently-colonized (P) and transiently-colonized (T). The model by *Chamchod* et al. only considered U-C in their framework [22]; the model by *Kajita* et al. considered U-C-I in their framework [25]; and the model by *Barnes* et al. considered U-P-T in their framework [28]. The relative importance in the transmission contributed by infected and colonized agents was addressed in U-C-I framework.

To depict transmission dynamics, endogenous and exogenous transmission pathways were explicitly stated in intra-facility and inter-facility transmission models. For intra-facility transmission models, endogenous pathways mainly described pathways that resulted in residents’ infection in NHs [22, 23] or CFs [25, 26]. HCWs were considered as transient host in one study only [22], and were defined as vectors to transmit MRSA via contaminated hands with “contaminating” time being less than an hour. At present, no reviewed article in CFs included staff in the transmission pathways. Exogenous pathways focused on the imported colonized residents. For inter-facility transmission models, the transmission pathways focused on patient transfers between the same type (hospital-hospital movements) or different types (LTCF-hospital movement) of facilities (Table 1).

Some common assumptions were made across all reviewed articles. One common assumption made was homogeneous contact mixing within the facility [22, 23, 25, 26, 28–31]. Another common assumption was fixed patient transfer rates in either hospitals or LTCFs, reflecting these movements were performed on a regular basis [22, 23, 28–31].

## Discussion

The scientific contributions to MRSA epidemiology of the 10 mathematical models were reviewed and summarized. To exhaust literatures specific to this research area, the search was based on both PUBMED and Google search engine. NHs provided ideal reservoirs for potential MRSA outbreaks in hospitals [22, 28]. It is further highlighted the role of NHs in the increasing nosocomial MRSA prevalence and the need for appropriate interventions in NHs [29–31], including contact precaution and intervention for residents with different colonization state (transiently colonized vs persistently colonized). Community colonization in NHs was found to have a strong effect on nosocomial colonization rates when MRSA was endemic in the health care system [31]. The relatively small population in prisons compared to the general public was found to be insignificant to MRSA community transmission [26, 27]. There was no consensus over the course of disease progression among reviewed models, which were mainly featured with NH residents /CF inmates/ hospital patients as transmission pathways and based on unrealistic assumptions. Some parameters used by these models were outdated or unfitted.

The reviewed models provided a starting point for future model development for intra-facility and inter-facility MRSA transmission. Moreover, infection control implications, transmission pathways, improvements for future work and future research direction can be identified from these models.

### Implication of infection control

MRSA epidemiology in hospitals is different from that in non-hospital residential settings. Therefore, traditional infection control measures for MRSA used in the hospitals are unlikely to be ineffective or inadaptable for LTCFs and CFs. For example, the minimization of stay is a common intervention in hospital settings but is inappropriate for LTCFs and CFs. Future strategies for MRSA prevention should include admission screening in LTCFs and discharge screening in CFs and hospitals, as well as longitudinal screening for residents. Screening at admission is supported by findings that a significant factor associated with MRSA epidemic in NHs was the introduction of MRSA colonized residents [22]. Frequent movement of NH residents from hospitals was also found to contribute to MRSA prevalence of small population LTCFs with small facility size and low turnover rate [28]. This practice is particularly important for residents with recent antibiotics exposure, as recent antibiotics exposure significantly increased MRSA acquisition rate and strain-specific MRSA prevalence [23, 24]. In addition, the similar rate of releasing colonized individuals from CFs and hospitals to the community [27] and the impact of recidivism on MRSA incidence in CFs [26] highlight the importance of discharge screening in all these facilities. Regular screening on residents during their stay should also be a part of infection control policy, as suggested by hospital study results that patients with negative screening results at admission can subsequently be colonized [32–34]. Consideration should also be made on how to prioritize different intervention strategies imposed on individuals with different colonization states [30]. It is noted that screening results are usually available 2–3 days after the test. Before the screening results are available, it is pertinent to recognize the use of isolation measures and their availability.

Other control measures include contact precautions and hand hygiene policy. Some hospital infection control guidelines [35] recommend contact precautions, including equipping staff with gowns and gloves. Preventing infected residents from entering cohorted rooms and equipping with protective clothing should be recommended for reduction of MRSA transmission in NHs [30]. Implementation of hand hygiene policy could have immense influence on the transfer rates of skin organism [36]. Inanimate items should be considered when considering suitable intervention strategies [37–39].

Difficulty in executing infection control policies varies. Screening and contact precaution require decisions at national, or at least institutional, level; while hand hygiene is more of facility or personal level and can be easily achieved through promotion. Therefore, implementing hand hygiene promotion while awaiting decisions for other policies shall be the optimal infection control schema.

### Transmission mechanisms of MRSA

Of the 10 reviewed studies, 9 of them considered residents/inmates as the only transmission pathway. Only one considered non-residents/non-inmates as a transmission pathway [22]. Prior study estimated MRSA carriage prevalence of 4.6% in 127 investigations among HCWs, and suggested that both transiently and persistently colonised HCWs were associated with several MRSA outbreaks involving few clusters [40]. Another study reported a colonization rate of 6.9% among nursing staff in non-outbreak situations [41]. None of the three reviewed models for CFs considered staff as a transmission pathway. The role of staff would need to be considered in future models as the number of staff present in LTCFs and CFs is likely to increase MRSA colonization or infection rates among residents.

Environmental contaminated objects, facility visitors and antibiotics resistance development were not considered in the 10 reviewed studies. Environmental contaminated objects were reported as a source of MRSA transmission [42]. Environmental intervention strategies have also been supported by modelling studies in hospital settings [43–46]. Environmental pathway had been shown to be important for crowded settings with limited hygiene resources, such as CFs and LTCFs [47–49]. Visitors’ role in MRSA transmission in hospital settings had been suggested as being significant pathways in non-hospital settings [50]. Besides, appearance of MRSA in the community may also increase the risk of MRSA carriage transfer from visitors to individuals in LTCFs or CFs [51]. Patients’ antibiotics exposure was found to be an important pathway by modelling studies in hospital settings [52] and by current reviewed studies [23, 24]. Future models for non-hospital facilities need to take these three pathways into consideration to increase their results applicability reflecting practical situations in the facilities.

The relative importance of different transmission modes was not considered in all 10 reviewed models. *Chamchod* et al. considered transmission via hands of HCWs [22]. However, it was also reported that: HCWs were contaminated with MRSA on their bodies [53]; An increase in MRSA-containing particles was shown in the air during and after bedmaking [54] or when the carriers suffered from upper respiratory tract infection [55]. Therefore, although transmission via contact is still considered as the main mode of transmission, the potential for other modes should be not eliminated.

Another element to be considered when building MRSA transmission models for non-hospital residential facilities is facility-specific characteristics, including: (a) LOS; (b) setting-specific risk factors; and (c) geographical and cultural differences. Previous studies found that LOS is a significant mediator of hospital-acquired MRSA [56]. This factor is not considered in the 10 reviewed models. Young age is a setting-specific risk factor for CFs [57]. This setting-specific risk factor can be utilized to develop age-structured model [58] for studying age-specific transmission patterns in CFs. Geographical and cultural differences in each type of facility may also impact transmission patterns. Factors such as climates, social practices and cultural behaviors may possibly act as mediators in transmission dynamics.

The ecological dynamics of MRSA strains were not well addressed in the reviewed models. Despite the hypervirulence possessed by strains being to the USA300 clone compared to other MRSA strains [59, 60], 8 of 10 models in this review did not differentiate the clones of MRSA under investigation. Only two recent models considered the transition of infections between USA300 and non-USA300 clones allowed an explicit niche of co-existence for these clones [23, 24]. However, the degree of competition for colonization of susceptible hosts or constructive interference of strains was not quantified [61].

### Model deficiencies

Some technical modelling execution deficiencies were listed in Additional file 1, Additional file 2 and Additional file 3. Empirical data used for parameterizing models were not updated. Empirical data for CFs is inadequate: there is no data available to parametrize the average decolonization duration of inmates, the proportion of colonized individuals progressing to infection and the average daily number of contacts in a CF [25]. In the inter-facility model by *Lesosky* et al. [31], the hospital colonization status parameters used were from the proportion of community MRSA positivity in 1998 and 2001–2004. Some of the reviewed studies did not state the years of data source and the parameter estimation procedures in their mathematical models (Additional file 1, Additional file 2 and Additional file 3).

Homogeneous social contact mixing was assumed in 8 of the 10 reviewed models [22, 23, 25, 26, 28–31]. This assumption would be unrealistic in many situations. Contact patterns have been found to be assortative with age in the general population [62]. The contact pattern within age groups was shown to be a key driver of age-specific infection rates [63]. Patterns of social contact mixing are greatly disparate across individuals in healthcare settings [64, 65]. Various health conditions of NH residents may cause their social contact mixing to be significantly more heterogeneous than the general population. It has also been found that CF inmates often intended to have low number of contact [25], but social contact among CF inmates was also expected to be heterogeneous. These factors should be incorporated into future models.

Almost all existing models are built explicitly for or informed by data from the US (Table 1). The existence of MRSA in non-hospital residential settings outside the US [66, 67] and the existence of geographic discrepancy in MRSA epidemiology [66] suggest there is a need for future modelling in other regions, such as Asia and Europe.

### Directions forward

Based on the transmission mechanisms of MRSA and examination of current model deficiencies, a list of recommendations for future mathematical transmission model development was composed (Additional file 4).

An evaluation of ongoing approaches by the latest MRSA modelling researches on hospitals shed light on future directions of MRSA modelling researches for non-hospital residential facilities in three perspectives: 1) infection control [28, 32, 68–73]; 2) ecology [74]; and 3) economy [27] (Additional file 5).

Future models should be ready to face the projected challenges from changes in population structure. The expected doubling of people over 60 by 2050 [75] put escalating demand for LTCFs. Decreasing average resident living area in the facilities will affect MRSA prevalence in LTCFs. In addition, the wide varieties of LTCFs, such as child day care center [76], suggest more modeling work is needed before robust conclusions can be drawn.

## Conclusions

Modelling studies on this important topic are at their initial phase of development and we identified critical opportunities for future work although many mathematical frameworks [77, 78] have been developed in previous studies. Importance of NHs has been highlighted from these current few studies addressing scattered aspects of MRSA epidemiology. However, the wide varieties of non-hospital residential settings suggest more work is needed before robust conclusions can be drawn. Learning from existing work for hospitals, we identified important future research direction in this area from infection control, ecological and economic perspectives. From current model deficiencies, we suggest more transmission pathways be specified to depict MRSA transmission, and further empirical studies should be stressed to support evidence-based mathematical models of MRSA in non-hospital facilities. Future models should also be ready to cope with the aging population structure.

## Additional files

Additional file 1: Technical model execution details for models in NHs. (DOCX 25 kb)

Additional file 2: Technical model execution details for models in CFs. (DOCX 26 kb)

Additional file 3: Technical model execution details for inter-facility models. (DOCX 36 kb)

Additional file 4: Recommendations for the future mathematical transmission model development based on A) Transmission mechanisms of MRSA; and B) Current model deficiencies. (DOCX 25 kb)

Additional file 5: Future directions of MRSA modelling researches for non-hospital residential facilities from 3 perspectives. (DOCX 20 kb)

## Abbreviations

ALFs: Assisted living facilities
C: Colonized
CFs: Correctional facilities
HCWs: Health-care workers
I: Infected
LOS: Length of stay
LTCFs: Long-term care facilities
MRSA: Methicillin-resistant *Staphylococcus aureus*
NHs: Nursing homes
P: Persistently-colonized
*S. aureus*: *Staphylococcus aureus*
T: Transiently-colonized
U: Uncolonized
