# Supplementary material for: A systematic review of transmission dynamic studies of methicillin-resistant Staphylococcus aureus in non-hospital residential facilities
Source: BMC Infect Dis. 2018 Apr 18;18:188. doi: 10.1186/s12879-018-3060-6 (PMC5907171; doi:10.1186/s12879-018-3060-6)
Supplement: Supplementary file 1 — Technical model execution details for models in NHs. (DOCX 25 kb) [file 12879_2018_3060_MOESM1_ESM.docx]

Additional file 1. Technical model execution details for models in NHs.

|  | Nursing Homes | | |
| --- | --- | --- | --- |
| **Execution details** | Chamchod et al. (2012) [22] | Batina et al. (2016a) [23] | Batina et al. (2016b) [24] |
| **Assumptions** |  |  |  |
| Mass-action | Yes (residents to residents, residents to HCWs, HCWs to residents) ^1^ | Yes (residents to residents) | Not applicable ^2^ |
| Homogenous contact mixing | Yes  ( residents to residents, residents to HCWs) ^1^ | Yes (residents to residents) | Not applicable ^2^ |
| Constant transmission rates | Yes | Yes | Yes ^3^ |
| Same admission and discharge rate | Yes | Yes | Not applicable ^2^ |
|  |  |  |  |
| **Parameter values** ^5^ |  |  |  |
| Transmission coefficient  (A) x (B) | 0.015 (residents to residents) 0.12 (HCWs to residents) ^1^  0.12 (residents to HCWs) ^1^ | 0.029/0.002/0.042 (per unit time)  (non-USA300; general/no AB/AB) 0.006/0.004/0.009 (per unit time) (USA300; general/no AB/AB) | Not applicable |
| Transmission probability (A) | 0.015 (residents to residents) 0.015 (HCWs to residents) ^1^ 0.015 (residents to HCWs) ^1^ | Not applicable | Not applicable |
| Contact rate (B) | 1 (daily number of contacts among residents) 8 (daily number of HCWs required per resident)^1^ | Not applicable | Not applicable |
| Transition probability | Not applicable | Not applicable | 0.913 - 0.984  (Susceptible to Susceptible) 0.222 - 0.333  (Colonized to Susceptible) 0.016 - 0.071  (Susceptible to Colonized) 0.011 - 0.716  (Colonized to Colonized) |
| Recovery rate of hosts | 1/80 - 1/60 (per day) | 0.116/0.125/0.107 (per unit time) (non-USA300; general/no AB/AB) 0.137/0.155/0.125 (per unit time) (USA300; general/no AB/AB) | Not applicable |
| Decontamination rate of vectors | 1 - 2 HCWs per hour | Not applicable | Not applicable |
| Facility size | 2000 | 446 (6 NHs combined as one population) | 446 (6 NHs combined as one population) |
| Probability of admission of colonized hosts | 0.1 | 0.215 | Not applicable |
| Resident-to-staff ratio | 3:1 / 4:1 | Not applicable | Not applicable |
| Admission rate | 1/365 resident per day | 0.074 (colonized residents per 3 months) 0.101 (uncolonized residents per 3 months) | Not applicable |
| Discharge rate | 1/365 resident per day | 0.074 (colonized residents per 3 months) 0.101 (uncolonized residents per 3 months) | Not applicable |
|  |  |  |  |
| **Ways of parameterization  (data source year, if stated)** |  |  |  |
| Empirical study | Yes (1991 - 2006 /2009^4^ ) | Yes (2008-2010) | Yes (2008-2010) |
| Adapted from old models | Yes (1995, 2007^4^, 2009^4^) | No | No |
|  |  |  |  |
| **Remarks** | | | |
| ^1^ HCWs were either contaminated or decontaminated but not MRSA carriers. | | | |
| ^2^ Pathway was not explicitly stated in this model, the probability of individual MRSA colonization state at time t had reflected the present amount of colonized in the facilities and individual current MRSA status. The current state at time t was assumed to be only dependent on their states at time t-1 | | | |
| ^3^ Same transition probabilities were used all the time. | | | |
| ^4^ Publication year is used as proxy. | | | |
| ^5^ Interpretations of parameter values should fit the context of the original model, and they may not be directly comparable across models. | | | |
