# Supplementary material for: A systematic review of transmission dynamic studies of methicillin-resistant Staphylococcus aureus in non-hospital residential facilities
Source: BMC Infect Dis. 2018 Apr 18;18:188. doi: 10.1186/s12879-018-3060-6 (PMC5907171; doi:10.1186/s12879-018-3060-6)
Supplement: Supplementary file 2 — Technical model execution details for models in CFs. (DOCX 26 kb) [file 12879_2018_3060_MOESM2_ESM.docx]

Additional file 2. Technical model execution details for models in CFs.

|  | Correctional facilities | | |
| --- | --- | --- | --- |
| **Items to specify the models** | Hartley *et al.* (2006) [27] | Kajita *et al.* (2007) [25] | Beauparlant *et al.* (2016) [26] |
| **Assumptions** |  |  |  |
| Mass-action ^1^ | Not applicable | Yes | Yes |
| Homogenous contact mixing | Not applicable | Yes | Yes |
| Constant transmission rates | Not applicable | Yes (regardless of colonized/infected) | Yes (regardless of hosts) |
| Same admission and discharge rate | Not applicable | No | No |
|  |  |  |  |
| **Parameter values** ^2^ |  |  |  |
| Transmission coefficient (A) X (B) | Not applicable | 0.00005 - 0.075 (male)  0.00005 - 0.1 (female) | 0.01 / 0.04 |
| Transmission probability (A) | Not applicable | 1 x 10⁻⁵- 1.5 x 10⁻³ (male) ^3^ 1 x 10⁻⁵- 2 x 10⁻³ (female) ^3^ | 1 ^4^ |
| Contact rate (per day) (B) | Not applicable | 5-50 (number; male)  5-50 (number; female) | 0.01 / 0.04  (regardless of subpopulation) |
| Transition probability | Not applicable | Not applicable | Not applicable |
| Recovery rate of hosts (per day) | Not applicable | 1/120 - 1/30  (male / female) ^5^ | 1/45  (community / incarcerated) ^6^ |
| Decontamination rate of vectors | Not applicable | Not applicable | Not applicable |
| Facility size | 700 (hospital) 3098 (prison) | 16956 (male) 2200 (female) | 100000  (community, inmates, recidivists) |
| Probability of admission of colonized hosts | Not applicable | 8.80 x 10⁻⁵- 4.92 x 10⁻³ (male) ^7^ 4.43 x 10⁻⁴ - 7.77 x 10⁻³ (female) ^7^ | Not applicable ^8^ |
| Resident-to-staff ratio | Not applicable | Not applicable | Not applicable |
| Admission rate (daily) | Not applicable | 341 - 407 (number; male) 64 - 81 (number; female) | 1/10000 (community) 1/365 (recidivists) |
| Discharge rate (daily) | 1/5 (hospital)  1/27 (prison) | 1/50 - 1/42 (male) 1/34 - 1/27 (female) | 1/45 |
| Proportion of colonized individuals  progressing into infection | Not applicable | 0.1 - 0.3  (male / female) | Not applicable |
| Average time for colonized individuals to progress to infection (days) | Not applicable | 4 - 15  (male / female) | Not applicable |
| Probability of recidivism | Not applicable | Not applicable | 0.4 |
| Death rate (per day) | Not applicable | Not applicable | 1 / (80*365) |
| Rate of individuals reaching age of majority (person per day) | Not applicable | Not applicable | Number of individual in community at time t * death rate |
|  |  |  |  |
| **Ways of parameterization  (data source year, if stated)** |  |  |  |
| Official data | Yes | Yes | Yes |
| Empirical study | No | Yes (1998 - 2004) | Yes (2005 - 2010) |
| Expert opinion | No | Yes | No |
| Estimation | No | Yes | Yes |
| Adapted from old models | No | No | Yes |
| Remarks |  |  |  |
| ^1^ In terms of force of transmission, it was density-dependent as it depended on number of colonized/infected individuals and proportion of susceptible individuals in the system. | | | |
| ^2^ Interpretations of parameter values should fit the context of the original model, and they may not be directly comparable across models. | | | |
| ^3^ Assumed the same for the flow of non-carriers being turned into colonized by both colonized and infected individuals. | | | |
| ^4^ Judged from the model formula. | | | |
| ^5^ From colonized to susceptible | | | |
| ^6^ From infected to susceptible | | | |
| ^7^ Assumed the same for importation of colonized / infected inmates | | | |
| ^8^ Separate compartments were set for community (infected) and recidivism (infected) to feature the flows between infected individuals. | | | |
