# Supplementary material for: A systematic review of transmission dynamic studies of methicillin-resistant Staphylococcus aureus in non-hospital residential facilities
Source: BMC Infect Dis. 2018 Apr 18;18:188. doi: 10.1186/s12879-018-3060-6 (PMC5907171; doi:10.1186/s12879-018-3060-6)
Supplement: Supplementary file 3 — Technical model execution details for inter-facility models. (DOCX 36 kb) [file 12879_2018_3060_MOESM3_ESM.docx]

Additional file 3. Technical model execution details for inter-facility models.

| **Items to specify the models** | Barnes et al. (2011) [28] | Lesosky et al. (2011) [31] | Lee et al. (2013a) [29] Lee et al. (2013b) [30] |
| --- | --- | --- | --- |
| **Assumptions** |  |  |  |
| Homogenous contact mixing | Yes | Yes | Yes ^13^ |
| Regular patient movement | Yes | Yes | Yes |
| Stratification of carriage type | Yes^2^ | No | Yes^14^ |
|  |  |  |  |
| **Parameter values**^1,7^ |  |  |  |
| Transmission coefficient |  |  |  |
| Hospitals | Hospitals: 0.15/0.25/0.35 Hospital units: 0.15/0.25/0.35 | N (0.00002, 0.000005) ^8^ x2 times ,x10 times | 0.0017 (0-0.2966) ^15^ |
| NHs/LTCFs | LTCF: 0.05/0.075/0.1 | N (0.00002, 0.000005) ^8^ x2 times ,x10 times | 0.000068 (0 - 0.0003) |
| MRSA prevalence (%) |  |  |  |
| Hospitals | Not applicable ^3^ | Not applicable ^3^ | 3.4 (1.1 - 18.5) |
| NHs / LTCFs | Not applicable ^3^ | Not applicable ^3^ | 25.9 (0 - 52) |
| Number of facility |  |  |  |
| Hospitals | 1 or more | Teaching hospitals (TH): 6 ; Non-teaching hospitals (NTH): 12 | 29 |
| NHs/LTCFs | 1 or more | 132 | 71 |
| Facility size |  |  |  |
| Hospitals | Hospitals: 300  Hospital units: 20 | TH: 460 ; NTH: 260 | 194 (48-505) |
| NHs/LTCFs | 100 | 155 | 99 (9-300) |
| LOS in the facility |  |  |  |
| Hospitals | Not applicable ^4^ | Normal patients^9^: Home: logNormal (0.68, 1.2) Transfer: logNormal (0.73, 2.21) Death: logNormal (0.8, 2.04) MRSA-positive patients^9^: Home: logNormal (0.95, 1.83) Transfer: logNormal (0.98, 2.85) Death: logNormal (0.98, 2.68) | Normal patients:  4 (1-626) (days) MRSA-positive patients:  8 (1- 414) (days) |
| NHs/LTCFs | Not applicable ^4^ | Not applicable | 37 (1-5066) (days) |
| Inflow and outflow of facility |  |  |  |
| Hospitals |  |  |  |
| Admission | Hospitals: 0.2 Hospital units: 0.05 / 0.2  (rate; time step) | Not applicable | 7033 (425 - 27151)  (number; annual) |
| Discharge | Hospitals: 0.2 Hospital units: 0.05 / 0.2  (rate; time step) | Transfer^10^: 0.035 / 0.07 / 0.14 Death^10^: 0.03 Home^10^: 0.83 / 0.9 / 0.935 | Community:  2699 (134 -16541) (number) |
| NHs/LTCFs |  |  |  |
| Admission | 0.002  (rate; time step) | Not applicable | 311 (3 - 7080)  (number; annual) |
| Discharge | 0.002  (rate; time step) | Transfer^11^: 0.4 Death^11^: 0.2  (rate; annual) | Community:  333 (17 - 1172) ^11^  (number) |
| Transfer |  |  |  |
| Hospitals to hospitals | Not applicable^5^ | TH - TH: 0.3 ^12^ TH - NTH: 0.2 ^12^ NTH - TH: 0.4 ^12^ NTH - NTH: 0.1 ^12^ | 80 (17 - 261) ^16^ |
| Hospitals to NHs / LTCFs | Associated weight^6^ | TH - NH: 0.5 ^12^ NTH - NH: 0.5 ^12^ | 679 (38.5 - 2616) ^16^ |
| NHs / LTCFs to Hospitals | Associated weight^6^ | NH - TH: 0.3 ^12^ NH - NTH: 0.7 ^12^ | 58 (0 - 261) ^16^ |
| NHs / LTCFs to NHs / LTCFs | Not applicable | Not applicable | 8 (0 - 64) ^16^ |
| Max. number of transfer | Not applicable | 3 | Not applicable |
| Cluster scenarios   (number of cluster) | Not applicable | 1 ( 2 / 3 / 6 ) | Not applicable |
| Re-admission to hospitals |  |  |  |
| Hospitals to hospitals  (number) | Not applicable^17^ | Not applicable^17^ | 1810 (82 - 7178) |
| NHs to Hospitals (number) | Not applicable^17^ | Not applicable^17^ | 249 (19 - 1403) |
| Time to readmission   (Hospitals to hospitals) (days) | Not applicable^17^ | Not applicable^17^ | 52 (1 - 366) |
| Time to readmission   (NHs to Hospitals) (days) | Not applicable^17^ | Not applicable^17^ | 50 (1 - 366) |
| Brief hospitalization |  |  |  |
| Number from NHs | Not applicable^17^ | Not applicable^17^ | 248 (0 - 1584) |
| LOS in hospitals (days) | Not applicable^17^ | Not applicable^17^ | 5 ( 0 - 14) |
| Average daily census |  |  |  |
| Hospitals | Not applicable | Not applicable | 103 ( 16 - 368) |
| NHs / LTCFs | Not applicable | Not applicable | 85.3 (9 - 214) |
| **Ways of parameterization  (data source year, if stated)** |  |  |  |
| Official data | No | Yes | Yes (2007) |
| Empirical study | Yes | Yes (1998, 2001-2004, not-specified) | Yes (2008-2009) |
| Expert opinion | No | No | No |
| Estimation | No | Yes | Yes |
| Adapted from old models | No | No | No |
| Remarks |  |  |  |
| ^1^ Values for [29,30] are expressed as median (range) for all 29 hospitals and 71 NHs. | | | |
| ^2^ MRSA carriage was further classified as "persistently colonized" and "temporary colonized" as defined in the compartmental framework inside a facility. | | | |
| ^3^ It is model outcome. For [31], MRSA prevalence in the community is: 2% (0.1, 4, 10). | | | |
| ^4^ Stay in the facility had been determined by admission and discharge rate | | | |
| ^5^ Number of hospitals was not explicitly stated in the article. In case of one hospital, it is not applicable. In case of more than one hospital, transfer between hospitals is determined by associated weight ^6^. | | | |
| ^6^ Intensity of patient transfer was determined by facility size, while proportion of patient in each disease state in a facility determined the types of individuals to be transferred.  Proportion of persistently colonized admitted patients among colonized patients to : hospitals / hospital units = 0 ; LTCF = 0.6 Proportion of transiently colonized admitted patients among colonized patients to : hospitals / hospital units = 1 ; LTCF = 0.4 Proportion of uncolonized admitted patients to : hospitals / hospital units = 0.9 ; LTCF = 0.9 | | | |
| ^7^ Interpretations of parameter values should fit the context of the original model, and they may not be directly comparable across models. | | | |
| ^8^  It was denoted as "transmission probability" in the text, but it incorporated a contact probability and a true disease transmission probability. So it was in fact "transmission coefficient". | | | |
| ^9^ LOS among those who discharged because of going home, transfer and death respectively. | | | |
| ^10^ Outflow of facility had been driven by the LOS distribution in the facility. They are the probabilities of being assigned different discharge outcomes among those discharged from hospitals. For Home, it referred to the original nursing homes or the community. | | | |
| ^11^ Residents in NHs can only leave NHs by death or transfer to hospitals in [31]. While in [29,30], residents in NHs can be transferred (to another NH or hospital) or to the community. | | | |
| ^12^ It is the probability of being transferred to different destinations among transferred agents. For agents from NHs/LTCFs to hospitals, the outcome of their next transfers could either be death or NHs/LTCFs. | | | |
| ^13^ Homogenous mixing within ward, but not across ward | | | |
| ^14^ 1/3 carriers had indefinite carriage; 2/3 carriers had a linear carriage loss with a half-life of 6 months. | | | |
| ^15^ Incidence of MRSA acquisition from ICUs and general wards were 3% and 1% respectively [29,30] and 2% for LTAC ward [30] under this parameter | | | |
| ^16^  Number of direct transfers | | | |
| ^17^ The mechanisms of readmission to hospitals or brief hospitalizations were implicitly included in the above parameters. | | | |
