# Supplementary material for: A systematic review of transmission dynamic studies of methicillin-resistant Staphylococcus aureus in non-hospital residential facilities
Source: BMC Infect Dis. 2018 Apr 18;18:188. doi: 10.1186/s12879-018-3060-6 (PMC5907171; doi:10.1186/s12879-018-3060-6)
Supplement: Supplementary file 4 — Recommendations for the future mathematical transmission model development based on A) Transmission mechanisms of MRSA; and B) Current model deficiencies. (DOCX 25 kb) [file 12879_2018_3060_MOESM4_ESM.docx]

**Additional file 4 Recommendations for the future mathematical transmission model development based on: A) Transmission mechanisms of MRSA; and B) Current model deficiencies.**

Citations here refer to the references section in this document.

1. Recommendations based on transmission mechanisms of MRSA

- Consider facility staff, environmental contaminated objects, facility visitors and antibiotics resistance development in the facility as transmission pathways;
- Consider the relative importance of different transmission pathways, such as staff and contaminated object;
- Consider relative importance of different transmission modes, such as touching and airborne dispersal;
- Consider facility-specific characteristics, such as LOS, setting-specific risk factors, and geographical and cultural differences;
- Consider the ecological dynamics of MRSA strains, such as the competition of hosts among MRSA clones.

1. Recommendations based on current model deficiencies

- Update empirical data to estimate model parameters;
- Incorporate heterogeneous social contact mixing pattern into the model;
- Collect currently missing empirical data to estimate model parameters.
- Quantify individual MRSA bacterial load and shedding in various disease states. Empirical work from this area provide better estimates involving MRSA disease course in non-hospital residential facilities, such as probability of colonized individuals developing infection and duration of decolonization. Previous empirical studies estimated the duration of MRSA colonization for hospital-admitted individuals served as good references [[1-3](#_ENREF_1)].
- Other parameters include transmission rate within facilities and transfer rate between facilities in the models.

**References:**

1. Rogers C, Sharma A, Rimland D, Stafford C, Jernigan J, Satola S, Crispell E, Gaynes R: **Duration of colonization with methicillin-resistant Staphylococcus aureus in an acute care facility: a study to assess epidemiologic features**. *American journal of infection control* 2014, **42**(3):249-253.

2. Scanvic A, Denic L, Gaillon S, Giry P, Andremont A, Lucet JC: **Duration of colonization by methicillin-resistant Staphylococcus aureus after hospital discharge and risk factors for prolonged carriage**. *Clinical infectious diseases : an official publication of the Infectious Diseases Society of America* 2001, **32**(10):1393-1398.

3. Robicsek A, Beaumont JL, Peterson LR: **Duration of Colonization with Methicillin-Resistant Staphylococcus aureus**. *Clinical Infectious Diseases* 2009, **48**(7):910-913.
