# Supplementary material for: A systematic review of transmission dynamic studies of methicillin-resistant Staphylococcus aureus in non-hospital residential facilities
Source: BMC Infect Dis. 2018 Apr 18;18:188. doi: 10.1186/s12879-018-3060-6 (PMC5907171; doi:10.1186/s12879-018-3060-6)
Supplement: Supplementary file 5 — Future directions of MRSA modelling researches for non-hospital residential facilities from 3 perspectives. (DOCX 20 kb) [file 12879_2018_3060_MOESM5_ESM.docx]

Additional file 5. Future directions of MRSA modelling researches for non-hospital residential facilities from 3 perspectives

| **Infection Control** | **Ecology** | **Economy** |
| --- | --- | --- |
|  |  |  |
| Evaluation of effectiveness of the following strategies: screening [32,68], triage/source isolation [69], skin disinfection [70], hand hygiene [72] and patient referral patterns [28,71] and contact tracing [73]. | The effects of antibiotic pressure [74] and the co-existence of different MRSA clones on MRSA transmission should be explored | The disease burden [27] associated with MRSA in non-hospital residential settings should be quantified to allow for resource allocation. |
|  |  |  |
